# Scraps

**Published:** 1892-12

**Authors:** 


### SCRAPS

PICKED UP BY THE ASSISTANT-EDITOR.

**The Guild of St. Cecilia.**—At an entertainment given to the inmates of a lunatic asylum in Scotland, one of the items of the programme consisted of a toy-symphony played by several influential gentlemen upon toy-instruments. After the symphony was over, a clergyman who had taken part in it asked one of the inmates, "Well, John, how did you like the music?" "Oh, weel, sir," was the reply," "it's a guid thing that we're a' daft here!"

**Unjustifiable Vicarious Sacrifice.**— In an account of the birth of the Duc de Bourgogne, grandson of Louis XIV., in the *Curiosités Historiques* (p. 48), amid the description of the raptures of the splendid court assembled on the occasion, there is a casual mention of an incident affording a wonderful contrast to all this royal joy and magnificence. The attendant chief *accoucheur*, the celebrated Dr. Clement, to prevent suffering on the part of the mother (the Dauphine), applied to her person the skin of a sheep, newly flayed. To obtain this quite fresh, a butcher was engaged to skin the animal alive, in the adjoining room; and being anxious to offer the skin as quickly as possible to the doctor, he carried it into the chamber of the Dauphine, leaving the door open. The sheep, in its agony, followed him, and ran in bleeding and skinless among the shrieking crowd of courtiers and grandees.—*Cornhill Magazine.*

**Medical Philology** (IV).—The *Promptorium* gives the following:

ATTYR, fylthe. *Sanies.*

Mr. Way's note is:—

A. Sax. Atter, *venenum.* "This sore is full of matter, or ater; *purulentum*," HORM. Atter has the same sense in Norfolk at the present time, and Skinner mentions the word as commonly used in Lincolnshire.

As the book referred to by Mr. Way as "HORM" was not one of those of which I gave a short description with the first of these paragraphs in March, I append a note upon it.

HORMAN ... *Vulgaria.* This was an English-Latin phrase-book compiled by William Horman, who was Head Master of Eton. The first edition of the book was printed in 1519. It was reprinted in 1530, five years before Horman's death.

This word, which is now obsolete, or only occasionally met with in some dialects, appeared in Old and Middle English in various forms which are given in the *New English Dictionary*, and which present great diversity. It is easy to see its alliance with the modern German, *Eiter*, pus. The surgical significance of the word is obvious in the passage in Caxton's *Golden Legend*, in which it is said "kyrnellys and botches of his face . . . . ranne grete plente of blood and atter;" and in Coverdale's version of *Job* ii. 7, which reads, "and scraped of the etter off his sores." No quotation later than 1643 is given in the Dictionary.

**Medical Ethics: Then and Now.**—I here give some extracts, written more than 300 years apart, which illustrate one of the topics on which satirists of doctors love always to dwell.

THEN.

In 1564 was published the first known edition of "*A Dialogve bothe pleaſaunte and pietifull, wherein is a goodly regimente againſt the feuer Peſtilence with a conſolacion and comfort againſt death.* Newly corrected by willyam

Bulleyn, the autour thereof." *Antonius* is represented ill with the plague. He sends for *Medicus*, who goes "solempnely ridyng vpon his mule, with a side goune, a greate chaine of gold about his necke, his Apothicarie *Crispine*, . . . . . . and his little Lackey, a proper yong applesquire called *Pandarus*, whiche carrieth the keye of his Chamber with hym." A long interview takes place. The apothecary is much affected by the sick man's recital of a dream, and the following whisperings occur:

*Crispine.*—I will departe: his talke doeth so muche trouble mee ; mee thinke he doeth wounde my conscience. Also I will home, and caste awaie a greate number of rotten drugges wherewith I haue gotten muche money in deceiuyng the people. God forgiue mee !

*Medicus.*—The vicar of S. Fooles be your ghostly father. Are you so wise ? tary still with mee; let hym paie for your rotten drugges; for I may saye to you that he is almoste rotten alreadie hymself; me think your conscience is to much spiced with sodaine deuotion.

Much further time is spent with the sick man, and then the dialogue thus proceeds:

*Medicus.*—Is it not tyme to depart? We haue taried here verie long, but not without gaine.

*Crispine.*—Sir, I haue thought it a moneth since our commyng hether : you haue been sente for eight tymes this after noone, and twoo of your pacientes are dedde this daie.

*Medicus.*—That is no maruell, for who can hold that will awaie. I shall haue more worke then I can put my hande vnto. It is now a golden worlde with me, and with you also.

*Crispinus.*—God continue the same. I would thousandes were sicke, but I would haue none dedde but the beggers that doe trouble the world, and haue no money to paie. I praie you what thinke you of maister Antonius; shall he escape it or no?

*Medicus.*—I haue his plentifull rewarde, and money for you also. I haue had lōg talke with hym. But to bee plain with you, I thinke neuer to se hym again aliue. He was paste cure or I came to hym, and he could not skape; therefore I kepte hym with longe talke, but I spake but softly.

*Crispine.*—Then I perceiue your talke was vnprofitable to him. Yet I wrote it in a little paper booke in my hande.

*Medicus.*—Not vnprofitable if the Phisicion come in the beginnyng or augmentyng of the sicknesse. But in the full state of this sickness, it is most dangerous, because death will preuente it or it cometh to the declinacion. Oh, it is a strong poison if the Pestilence crepe to his harte.

*Crispine.*—This man loued you well in his life; if he dooe depart this present worlde, will ye not be present at his buriall, Maister doctour?

*Medicus.*—He loued me as I loued him, He me for healthe, and I hym for money; And thei whiche are preseruers of the life of manne, ought not to be present at the death or buriall of the same man, therefore I haue taken my leaue, I warrante you.

NOW.

On sait que ce sont les chirurgiens américains qui ont mis en honneur l'intervention précoce dans l'appendicite. Il parait qu'ils abusent un peu de ce diagnostic, du moins si l'on en croit un des journaux médicaux de Philadelphie, qui n'est pas tendre pour ses compatriotes.

Notre confrère américain réclame vivement au nom de la clinique et de l'honnêteté. "Il semble," dit-il, "que, dans les examens des étudiants en médecine, les questions habituelles sur le diagnostic différentiel de la constipation avec les autres formes d'obstruction intestinale aient été remplacées par les suivantes ou par quelque chose d'approchant : "

"Dites-moi les caractères différentiels des affections abdominales ?"

"Si le sujet est du sexe masculin, il a une appendicite ; si c'est une femme, elle a une salpingite."

"Quelle est l'indication de la laparotomie ? "

"L'aptitude du malade à payer la forte somme."

"Nous n'en sommes pas encore là pour l'appendicite," dit notre ami T. dans la *Médecine moderne* "mais pour la salpingite, je ne jurerais que parfois! . . . . ."—*L'Union médicale*, 1892.

